# Iron overloaded M0 macrophages regulate hematopoietic stem cell proliferation and senescence via the Nrf2/Keap1/HO-1 pathway

**DOI:** 10.1515/biol-2025-1206

**Published:** 2025-12-30

**Authors:** Lu Bai, Fan Wang, Hongmei Ouyang, Huixian Bai, Chenrong Wang, Jiaxin Liu

**Affiliations:** Department of Clinical Laboratory, The First People’s Hospital of Yunnan Province, The Affiliated Hospital of Kunming University of Science and Technology, Kunming, Yunnan Province, 650032, China; School of Medicine, Kunming University of Science and Technology, Kunming, Yunnan Province, 650500, China

**Keywords:** iron overload, macrophages, hematopoietic stem cells, Nrf2/Keap1/HO-1, TMC

## Abstract

Numerous studies have established a link between iron overload and hematological disorders, yet its impact on hematopoietic stem cell (HSCs) homeostasis remains unclear. This study investigates the effects of iron-overloaded macrophages on HSCs proliferation and senescence, focusing on the potential protective role of the Nrf2/Keap1/HO-1 signaling pathway. In this experiment, THP-1 cells were first differentiated into M0 macrophages, which were then exposed to ferric ammonium citrate (FAC) to establish an iron overload model. The impact of iron overload on macrophage function was assessed by measuring phagocytic activity, reactive oxygen species (ROS) levels, and inducible nitric oxide synthase (iNOS) expression. A co-culture system with HSCs was used to evaluate the effects of iron-overloaded macrophages on HSCs proliferation, cell cycle progression, and senescence. Western blot analysis was employed to measure oxidative stress and aging markers. Nrf2 activation was induced to assess its protective role. The results showed that iron overload significantly impaired macrophage function, as evidenced by reduced phagocytic activity, increased ROS production, and elevated iNOS expression. In the co-culture system, iron-overloaded macrophages inhibited HSCs proliferation, induced cell cycle arrest, and accelerated senescence, as evidenced by increased aging markers (P16, SA-β-gal) and decreased proliferative markers (HOXB4, RUNX1). Nrf2 activation with 2-trifluoromethyl-2′-methoxychalcone (TMC) reversed these effects, restoring HSCs proliferation and reducing oxidative damage. In conclusion, this study explores how iron-overloaded macrophages affect HSCs through the Nrf2/Keap1/HO-1 pathway.

## 1 Introduction

Macrophages are key components of the innate immune system. They maintain tissue homeostasis, regulate immune responses, and clear pathogens via phagocytosis and cytokine secretion [1], 2]. Their functional flexibility – spanning pro-inflammatory (M1) to anti-inflammatory (M2) phenotypes – is strictly controlled by environmental cues, including iron availability [3]. Iron is a redox-active micronutrient. It supports vital macrophage functions, such as enzyme activity and oxidative burst [4]. However, excess iron is harmful. It drives reactive oxygen species (ROS) production through Fenton chemistry and disrupts macrophage polarization [5]. In diseases like transfusion-dependent anemia, macrophages accumulate iron and switch to a cytotoxic M1 phenotype. This phenotype is characterized by high inducible nitric oxide synthase (iNOS) expression and reduced phagocytic ability, which may disturb the surrounding cellular microenvironment [6], 7].

In the bone marrow, hematopoietic stem cells (HSCs) rely on a precisely balanced niche for self-renewal and differentiation. Macrophages are now identified as key regulators of this niche [8]. However, iron-overloaded macrophages exhibit defective erythrophagocytosis and impaired iron export. These defects may disrupt HSCs quiescence and function [9]. Recent studies indicate that oxidative stress from these macrophages accelerates HSCs aging by weakening antioxidant defenses [10], 11]. Despite these findings, the specific mechanisms connecting macrophage iron overload to HSCs dysfunction remain unclear. This is especially true for the role of the Nrf2/Keap1/HO-1 pathway – a core regulator of cellular redox balance.

The Nrf2 pathway protects cells from oxidative damage. It does so by activating genes like HO-1, which enhance antioxidant responses [12]. Under normal conditions, Nrf2 activity in macrophages helps maintain HSCs by neutralizing ROS. But in iron-overloaded macrophages, this protective mechanism is impaired. Increased Keap1 promotes Nrf2 degradation and blocks its nuclear translocation. This leads to uncontrolled oxidative stress [13]. While ROS-induced HSCs dysfunction has been reported [14], it is still unknown whether this is specifically driven by Nrf2 suppression in macrophages, rather than intrinsic changes in HSCs. Additionally, it has not been systematically studied whether pharmacological activation of Nrf2 – for example, with 2-trifluoromethyl-2′-methoxychalcone (TMC) – can reverse these effects in the context of macrophage-HSCs interactions.

To answer these questions, we explored whether iron overload disrupts Nrf2/Keap1/HO-1 signaling in macrophages. We hypothesized this disruption would promote M1 polarization and oxidative stress, thereby impairing HSCs function. Using an *in vitro* co-culture system, we found that iron-loaded macrophages reduce nuclear Nrf2, increase Keap1 expression, and lower HO-1 levels. These changes were associated with HSCs cycle arrest and elevated senescence markers (P16, SA-β-gal). Importantly, TMC treatment reactivated Nrf2 signaling in macrophages. It also restored HSCs proliferation and viability. These results identify macrophage Nrf2 signaling as a critical mediator of iron-induced hematopoietic dysfunction. They also highlight the therapeutic potential of targeted Nrf2 activation.

## 2 Materials and methods

### 2.1 Cell culture conditions

The human monocytic leukemia cell line THP-1 (Procell Life Science, CL-0233) was cultured in RPMI-1640 medium (Procell Life Science, PM150110), supplemented with 10 % fetal bovine serum (FBS; Gibco, 10099141) and 1 % penicillin-streptomycin (New Cell & Molecular Biotech, C100C5). Cells were maintained in a 37 °C incubator with a 5 % CO_2_ atmosphere and passaged regularly to maintain exponential growth. Rat bone marrow-derived hematopoietic stem cells (HSCs) (Zhong Qiao Xin Zhou Biotechnology, PRI-RAT-00152) were cultured in complete HSCs medium (Zhong Qiao Xin Zhou Biotechnology, PCM-R-152) under the same culture conditions (37 °C, 5 % CO_2_). The medium for HSCs was refreshed every two to three days to ensure optimal cell growth and viability. Both THP-1 cells and HSCs were monitored regularly for contamination and morphology.

**Informed consent:** Not applicable.

**Ethical approval:** Not applicable.

### 2.2 Differentiation and validation of M0 macrophages

To induce M0 macrophage differentiation, THP-1 cells were treated with 50 μg/mL phorbol 12-myristate 13-acetate (PMA; MedChemExpress, HY-18739) dissolved in 0.1 % dimethyl sulfoxide (DMSO) for 24 h. Control cells received equivalent volumes of DMSO (0.1 % v/v). Differentiation was confirmed through multiple assays: cell viability was assessed using the CCK-8 assay (Biosharp, BS350A), with absorbance measured at 450 nm. Phagocytic activity was evaluated by neutral red uptake method (Solarbio, G1316), with absorbance quantified at 540 nm. Bright-field microscopy (AE2000, Motic) examined the cells morphology. Expression of surface marker CD11b and CD68 observed by immunofluorescence staining (Nikon T2 fluorescence microscope) using anti-CD11b (Proteintech, 65116-1-lg) and anti-CD68 (Proteintech, 65187-1-lg) antibodies.

### 2.3 Iron overloaded modeling and validation

In order to construct iron-overloaded M0 macrophages, we first set up several groups of ferric ammonium citrate (FAC; MedChemExpress, HY-B1645), such as 10, 20, 40, 80, 160 μmol/L in THP-1 cells induced by PMA and cultured for 24 h. Control (THP-1 + PMA group) was received equivalent volumes of phosphate-buffered saline (PBS). After the FAC treatment, cell viability was assessed using the CCK-8 assay with absorbance at 450 nm. By comparing the OD450 values between the FAC treatment group and the control group, the effect of FAC concentration on cell viability was analyzed, and the appropriate FAC concentration for constructing iron overload model was determined.

To verify the successful construction of the model, different concentrations of FAC (10, 20, 40, 80, 160 μmol/L) were added after PMA induced M0 macrophages with THP-1 were continued for 24 h. After treatment, Calcein-AM fluorescent probe (MedChemExpress, HY-D0041; excitation/emission: 494/515 nm) was added, and after incubation for a period of time, fluorescence microscope was used to observe and record the intracellular fluorescence intensity. The change of fluorescence intensity reflected the change of the concentration of variable iron pool in the cell, so as to verify whether the cell successfully constructed the iron overload model.

### 2.4 ROS detection by immunofluorescence

M0 macrophages treated with 160 µM FAC were used to construct the iron-overloaded macrophage model. Cells were seeded onto Petri dishes with pre-placed coverslips and cultured until they adhered and reached an optimal growth state.

To preserve cellular morphology, cells were fixed with 4 % paraformaldehyde for 15 min, followed by permeabilization with 0.1 % Triton X-100 for 5 min to facilitate the entry of fluorescent probes. Non-specific binding was minimized by blocking with PBS containing 5 % bovine serum albumin (BSA) for 30 min. ROS immunofluorescence detection reagent was added according to the manufacturer’s instructions, and cells were incubated at appropriate temperature and humidity for 1 h. The fluorescent probe specifically bound to intracellular ROS. After incubation, cells were washed three times with PBS for 5 min each to remove unbound probes. Coverslips were then mounted onto glass slides using an anti-fade mounting medium, and fluorescence imaging was performed with an inverted fluorescence microscope. Fluorescence intensity was quantified using image analysis software. ROS levels in normal macrophages group and iron-overloaded macrophages group were compared to assess oxidative stress induced by iron overload.

### 2.5 Transwell co-culture system

All cell culture reagents and Transwell chambers (0.4 μm pore size, Corning, 3470) were pre-warmed to 37 °C. HSCs (5 × 10^4^ cells/well) were cultured in H-DMEM medium (Procell, PM150210B) supplemented with 10 % FBS and 1 % penicillin-streptomycin until reaching the logarithmic growth phase. The cells were then digested, washed twice with PBS, followed by a rinse with serum-free H-DMEM, resuspended in serum-free medium, counted, and adjusted to a final concentration of 2 × 10^5^ cells/mL.

For the Transwell co-culture, HSCs (5 × 10^4^ cells/well) were seeded in the lower chamber with 600 μL of complete H-DMEM medium (10 % FBS, 1 % penicillin-streptomycin). The plate was incubated at 37 °C for 24 h to allow cell adhesion. In the upper chamber, 100 μL of M0 macrophage suspension (2 × 10^5^ cells/mL in serum-free RPMI-1640 medium) was added. After the 24-h co-culture period, the Transwell chamber was carefully removed using forceps. The liquid from the upper chamber was blotted dry, and the chamber was transferred to a well containing approximately 800 μL of methanol for fixation at room temperature for 5 min.

Following fixation, the upper chamber was blotted dry again and transferred to a well containing approximately 800 μL of Giemsa staining solution. The chamber was stained at room temperature for 15 min. After staining, the chamber was gently rinsed and soaked with water multiple times. The liquid from the upper chamber was absorbed, and the cells on the membrane surface were carefully wiped with a wet cotton swab. The membrane was then carefully removed with small tweezers, left to dry with the bottom side up, and transferred to a glass slide. Finally, the membrane was sealed with neutral gum. Migrated cells were counted in five standardized fields (central and four quadrants) using ImageJ software.

### 2.6 Cell viability assay

Cell viability was evaluated using the CCK-8 assay, with absorbance measured at 450 nm. In the experimental process of inducing THP-1 to differentiate into M0 macrophages. THP-1 cells were differentiated into M0 macrophages by treating with 50 μg/mL PMA for 24 h. After differentiation, 10 µL of CCK-8 solution was added to each well of a 96-well plate, and the absorbance at 450 nm was measured after 4 h of incubation. This measured the viability of THP-1-differentiated macrophages, and the OD values were compared between the control and PMA-induced groups to assess the impact of PMA on cell viability.

When constructing *in vitro* models of iron-overloaded macrophages, M0 macrophages were treated with varying concentrations of FAC (10–7,780 μM) for 4 h. CCK-8 assays were performed to determine cell viability, with OD values at 450 nm compared between the 160 μM FAC-treated and control (THP-1 + PMA) groups. The concentration of FAC that induced significant iron overload without adversely affecting cell viability was identified.

To study the effect of iron-overloaded macrophages on the survival and differentiation of HSCs, HSCs were co-cultured with either normal M0 macrophages, iron-overloaded M0 macrophages, or iron-overloaded M0 macrophages treated with 1 μM TMC, an Nrf2 activator, for 12 h. Co-culture was performed for 24 h. The viability of HSCs was assessed by CCK-8 assay, with OD values at 450 nm compared between the four groups: HSCs alone (HSCs), HSCs co-cultured with normal macrophages (HSCs + THP-1 + PMA), HSCs co-cultured with iron-overloaded macrophages (HSCs + THP-1 + PMA + FAC), HSCs co-cultured with iron-overloaded macrophages with Nrf2 activation (HSCs + THP-1+PMA + FAC + TMC).

### 2.7 Neutral red uptake assay

Phagocytosis activity was evaluated by neutral red uptake assay. In the experimental process of inducing THP-1 to differentiate into M0 macrophages. After PMA induction, THP-1 cells were incubated with a 0.1 % neutral red solution for 2 h. Following incubation, the cells were carefully washed with PBS 3 times to remove excess and non-internalized dye. The absorbed neutral red dye within the cells was then solubilized by adding a solubilization solution consisting of 50 % ethanol and 1 % glacial acetic acid (v/v) to each well. The plate was gently shaken at room temperature for 15 min to ensure complete dissolution of the dye.

Absorbance was thereafter measured at 540 nm (OD540) using a microplate reader. The OD values were compared between the untreated control and PMA-induced groups to evaluate the effect of PMA on the phagocytic activity of THP-1.

### 2.8 Western blot analysis

Total cellular proteins were extracted using radio-immunoprecipitation assay lysis buffer supplemented with protease and phosphatase inhibitors. The lysate was centrifuged at 12,000 revolutions per minute at 4 °C for 10 min, and the protein supernatant was collected. Protein concentration was determined by the bicinchoninic acid method.

After denaturing the samples with sodium dodecyl sulfate loading buffer, the samples were separated using sodium dodecyl sulfate-polyacrylamide gel electrophoresis, and then the proteins were transferred to a polyvinylidene Fluoride membrane. Following blocking of the membrane, specific primary antibodies – anti-iNOS antibody (1:2,000, ab15323), anti-Nrf2 antibody (1:2,000, ab62352), anti-Keap1 antibody (1:2,000, OTI1B4), anti-HO-1 antibody (1:2,000, EPR18161-128), anti-P16 antibody (1:2,000, EP1551Y), anti-HOXB4 antibody (1:2,000, EPR1917), anti-RUNX1 antibody (1:2,000, EPR3099), anti-β-actin antibody (1:2,000, mAbcam 8226) (all antibodys purchased from Abcam, Shanghai, China) were added and incubated overnight at 4 °C. After washing with Phosphate-Buffered Saline containing Tween-20 (PBST), the corresponding horseradish peroxidase-conjugated secondary antibodies were added. Protein bands were developed using an Enhanced Chemiluminescence detection kit (Proteintech, China). Images were captured with a chemiluminescence imaging system, and densitometric analysis was performed using ImageJ software to determine the gray values of the bands.

Each treatment group contained 3 independent samples, and the Western blot experiment was independently repeated 3 times to verify the results.

### 2.9 Flow cytometry analysis

Intracellular staining of Ki67 and DNA was performed according to previous studies [15]. Cells were fixed and permeabilized using Foxp3/Transcription Factor Staining Buffer (Affymetrix, eBioscience). Then, intracellular staining for Ki67 was performed. The primary antibody used was an anti-Ki67 monoclonal antibody (clone SolA-15; eBioscience, Thermo Fisher). It was conjugated to either fluorescein isothiocyanate. After staining, the cells were washed. They were then incubated with 2 μg/mL Hoechst 33342 (Thermo Fisher Scientific, Thermo Fisher) in PBS at room temperature for 15 min. This step was for DNA staining. Subsequently, the cells were centrifuged at 400×*g*. They were resuspended in PBS and analyzed by flow cytometry. For sample analysis, an LSRFortessa flow cytometer (BD Biosciences) was used. It was paired with the accompanying DIVA software. Finally, data analysis was completed using FlowJo software v.10.

### 2.10 SA-β-gal staining

SA-β-gal expression was assessed using a staining kit (Beyotime, C0602). The culture medium of HSCs, HSCs co-cultured with macrophages (THP-1 + PMA) and HSCs co-cultured with iron-overloaded macrophages (THP-1 + PMA + FAC) were removed and gently washed cells 3 times with PBS for 1 min each. Fix cells with 1 mL fixative for β-galactosidase staining for 15 min at room temperature. PBS was removed by aspiration, and 1 mL of staining working solution was added to each well. The cells were incubated overnight at 37 °C. The number of SA-β-gal-positive cells was quantified in five randomly selected fields using a Nikon Eclipse Ti microscope at 200× magnification.

In order to evaluate the regulatory role of Nrf2 activation in SA-β-gal expression, the culture medium of HSCs was co-cultured with iron-overloaded M0 macrophages treated with 1 μM TMC were also removed and washed by PBS, cells were fixed and stained with staining working solution.

### 2.11 Data analysis

Data are presented as mean ± standard deviation (SD). All statistical analyses and graph plotting were performed using GraphPad Prism 8.0 software. Prior to formal statistical testing, the Shapiro-Wilk test was applied to assess the normality of data distribution. For data that passed the normality test (*P* > 0.05), parametric tests were used: the unpaired student’s *t*-test was employed to compare differences between two groups (e.g., comparison between the control group and the PMA-treated group). One-way analysis of variance (ANOVA) was used, followed by the Tukey Honestly Significant Difference (HSD) post-hoc test to correct for multiple comparisons. Statistical significance was defined as *P < 0.05, **P < 0.01, ***P < 0.001, and ****P < 0.0001 to provide quantitative clarity on the strength of observed effects.

## 3 Results

### 3.1 PMA-induced differentiation of THP-1 cells into macrophages

**Figure 1:**
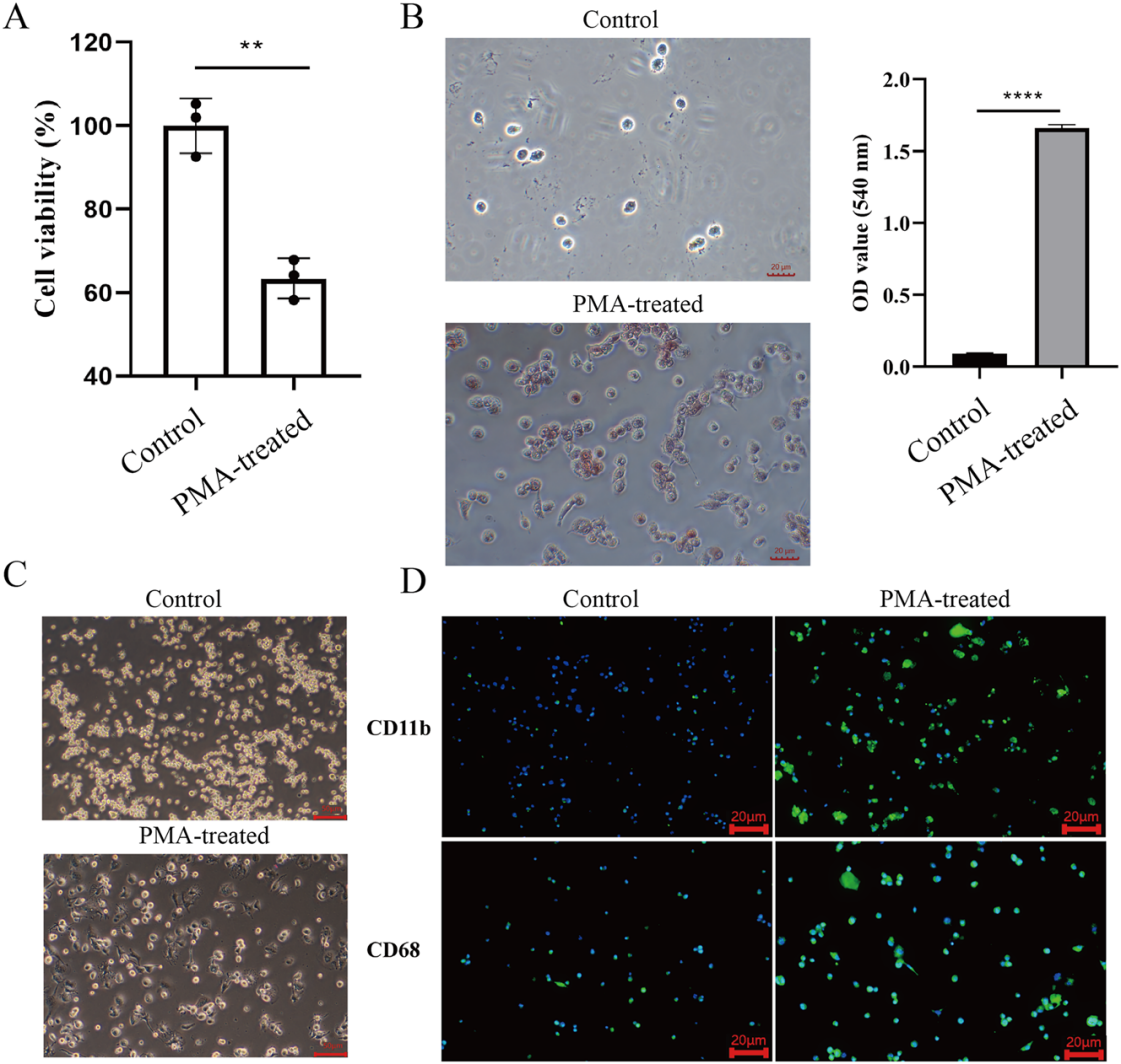
PMA-induced differentiation of THP-1 cells into M0 macrophages. (A) Cell viability was assessed using the CCK-8 assay at 450 nm (OD450). A significant reduction in viability was observed in the PMA-treated group (50 μg/mL) compared to the untreated control group (0 μg/mL). (B) Phagocytic activity was evaluated using the neutral red uptake assay at 540 nm (OD540), with a marked increase in absorbance in the PMA-treated group compared to the control group. (C) Bright-field microscopy images illustrating morphological changes in THP-1 cells following PMA treatment. (D) Immunofluorescence staining of macrophage markers CD11b and CD68 in THP-1 cells post-PMA treatment. Scale bar: 20 μm (*n* = 3; **P < 0.01, ****P < 0.0001).

To confirm the differentiation of THP-1 cells into M0 macrophages following PMA treatment, functional assays were performed. Cell viability, assessed via CCK-8 assay, showed a significant reduction in the PMA-treated group (50 μg/mL) compared to the untreated control (0 μg/mL) (*P* < 0.01) (Figure 1A). Phagocytic activity, evaluated using the neutral red uptake assay, was significantly enhanced in PMA-treated group relative to controls (*P* < 0.0001) (Figure 1B). Bright-field microscopy revealed distinct morphological changes post-PMA treatment. Control cells maintained a small, round monocyte-like appearance, whereas PMA-treated cells displayed increased size, irregular shapes, and an adherent phenotype, characteristic of differentiated M0 macrophages (Figure 1C). Immunofluorescence staining further confirmed differentiation by detecting macrophage surface markers. PMA treatment significantly upregulated CD11b and CD68 expression compared to the control, reinforcing successful macrophage differentiation (Figure 1D).

### 3.2 Establishment of an iron-overloaded M0 macrophage model

**Figure 2:**
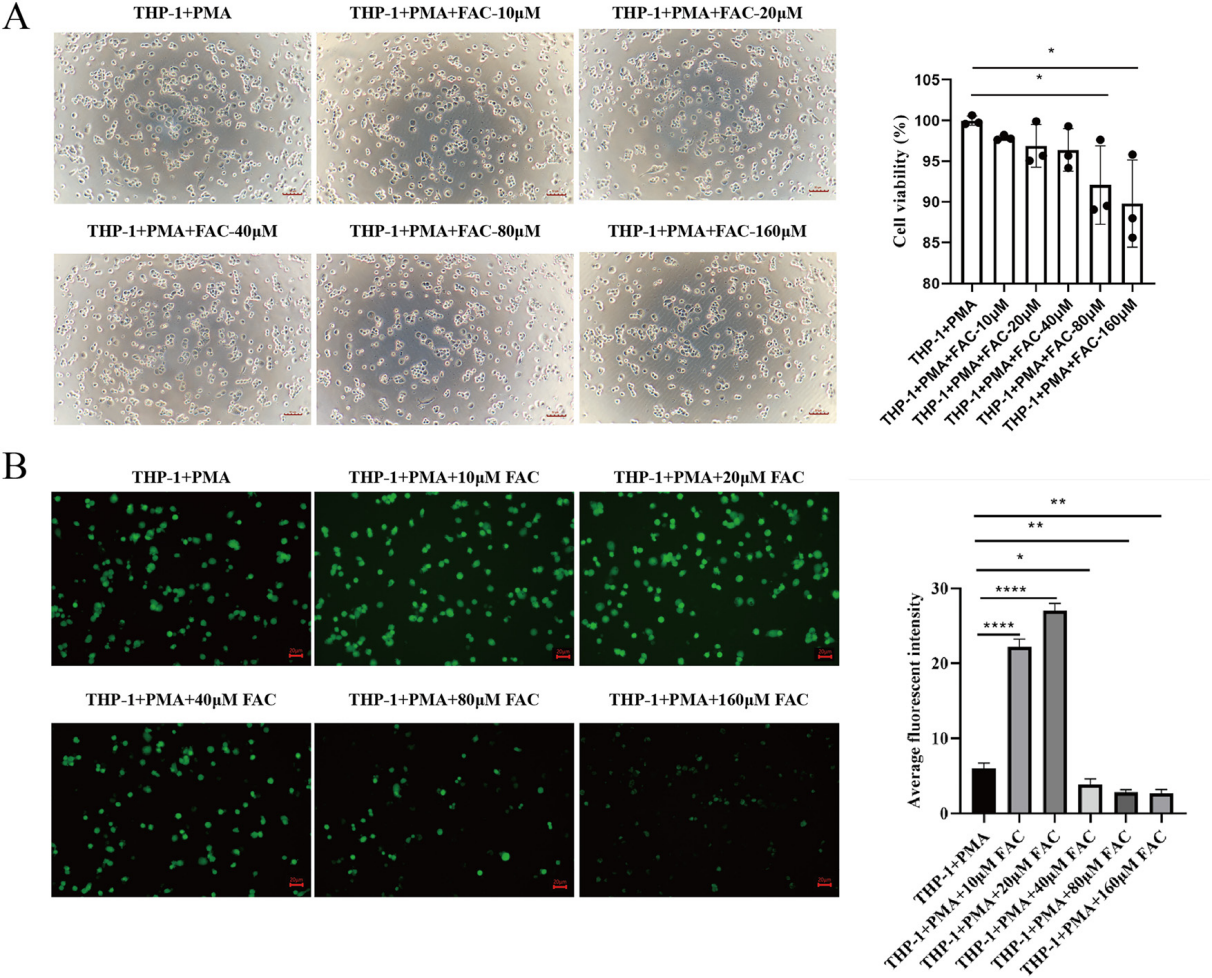
Effect of varying FAC concentrations on cell viability and intracellular iron accumulation. (A) Changes in cell viability after treatment with different concentrations of FAC (10, 20, 40, 80, and 160 µM). Scale bar: 50 µm. (B) Intracellular iron content after treatment with different concentrations of FAC. Scale bar: 20 μm (*n* = 3; *P < 0.05, **P < 0.01, ****P < 0.0001).

To establish an iron overload model in differentiated M0 macrophages, PMA-differentiated THP-1 cells were treated with various concentrations of FAC. Cell viability was assessed using the CCK-8 assay. As shown in Figure 2A, a slight decline in cell viability was observed with increasing FAC concentrations (10 µM–160 µM). Next, intracellular iron accumulation was measured by quantifying the LIP (Labile Iron Pool) using the Calcein-AM fluorescence assay. The results showed that compared with the untreated control group, cells treated with 10 μM and 20 μM FAC had significantly increased fluorescence intensity. In contrast, the fluorescence intensity of cells treated with 40 μM, 80 μM, and 160 μM FAC was significantly lower than that of the control group, with the 160 μM group showing the lowest value (Figure 2B). A lower mean fluorescence intensity directly indicates a higher LIP level, which further leads to more severe intracellular iron overload (Figure 2B). Based on these findings, a concentration of 160 µM FAC was selected for subsequent experiments, as it effectively induced iron overload in M0 macrophages.

### 3.3 Phenotypic changes in iron-overloaded M0 macrophages

**Figure 3:**
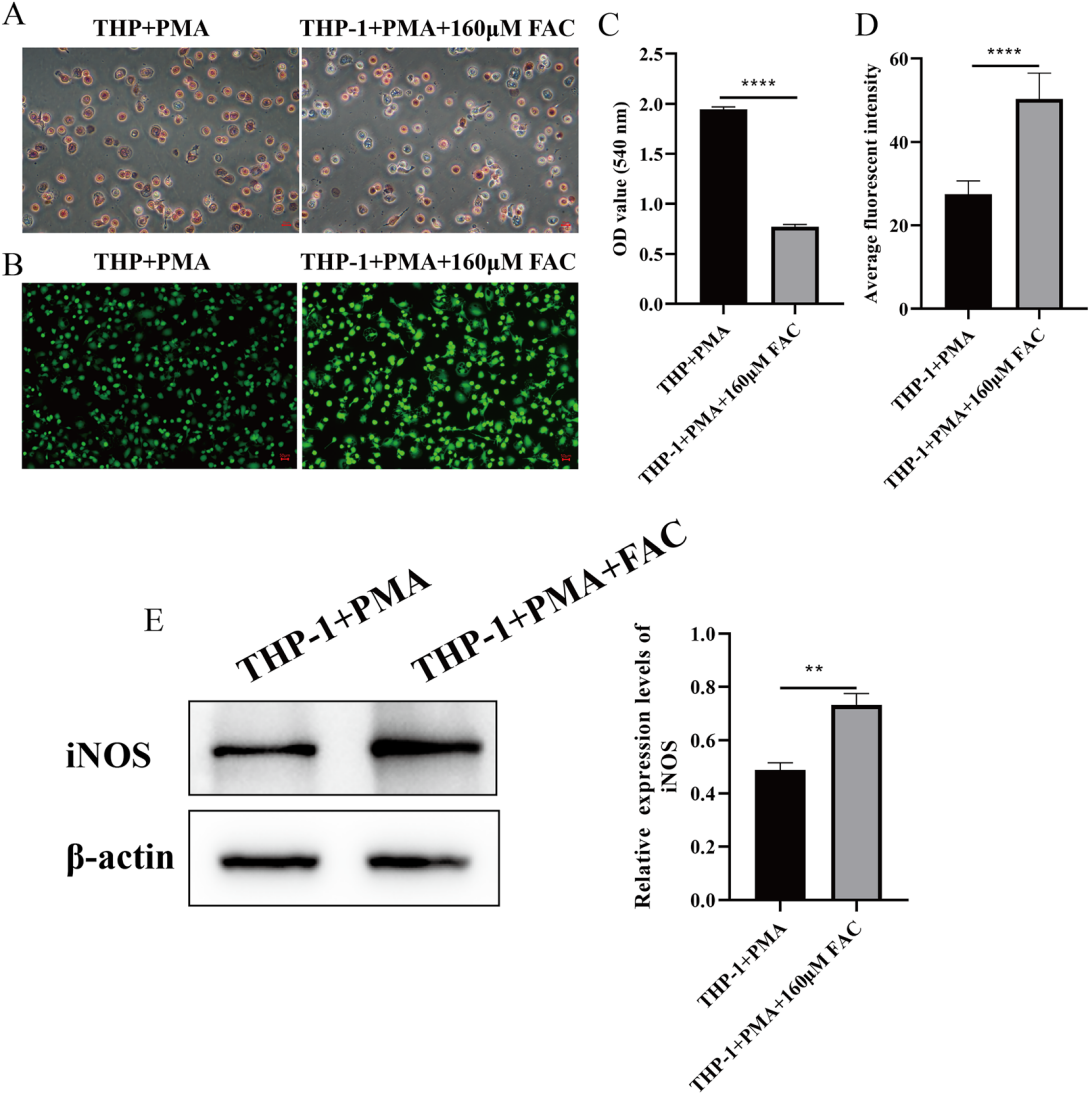
Phenotypic alterations in iron-overloaded M0 macrophages. (A/C) Phagocytic activity was assessed using the neutral red uptake assay, with absorbance measured at OD540. A significant reduction was observed in the iron-overloaded group (THP-1+PMA + FAC) compared to the control (THP-1+PMA). Scale bar: 20 μm. (B/D) ROS levels were evaluated via immunofluorescence staining. Quantification of fluorescence intensity revealed a significant increase in the iron-overloaded group. Scale bar: 50 µm. (E) Western blot analysis of iNOS expression, normalized to β-actin, demonstrated a significant upregulation in the iron-overloaded group. (*n* = 3; **P < 0.01, ****P < 0.0001).

To evaluate the effects of iron overload on macrophage function, an iron-overloaded M0 macrophage model was established by treating PMA-differentiated THP-1 cells with 160 µM FAC. Phagocytic activity, assessed via the neutral red uptake assay, was significantly reduced in iron-overloaded M0 macrophages compared to the untreated control (*P* < 0.0001) (Figure 3A/C). ROS levels were quantified using immunofluorescence staining, revealing a marked increase in fluorescence intensity in iron-overloaded macrophages (*P* < 0.0001) (Figure 3B/D). Western blot analysis further demonstrated a significant upregulation of iNOS, an inflammatory and oxidative stress marker, in the iron-overloaded group compared to the control (*P* < 0.01) (Figure 3E).

### 3.4 Phenotypic changes in HSCs induced by iron-overloaded macrophages

**Figure 4:**
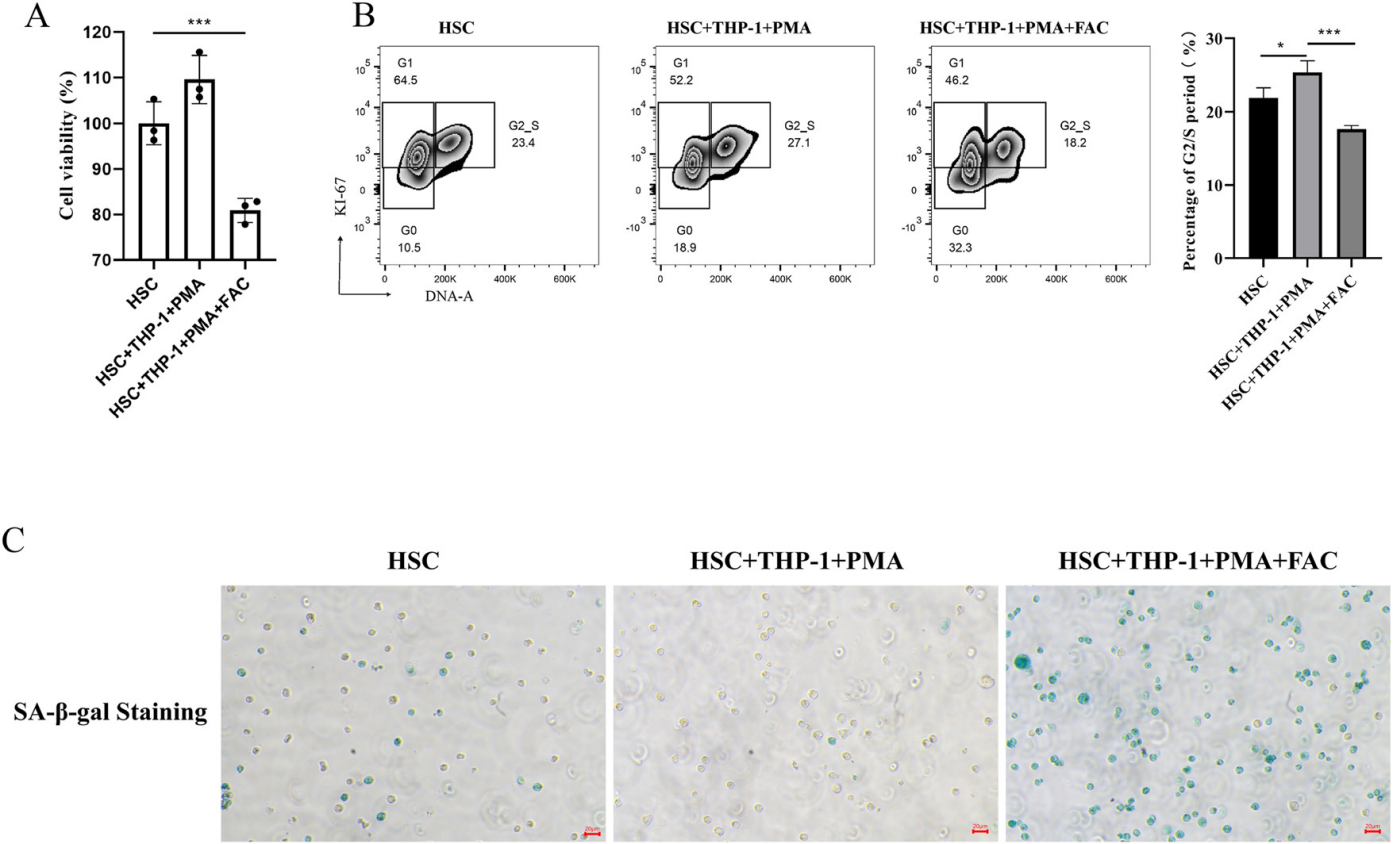
Phenotypic changes in HSCs induced by iron-overloaded M0 macrophages. (A) The cell viability was measured by CCK-8 assay in different groups. (B) Cell cycle progression was analyzed by flow cytometry, with statistical analysis showing the percentage of cells in the G2/S phase across different groups. (C) Representative images of SA-β-gal staining from different experimental groups. Scale bar: 20 μm (*n* = 3, *P < 0.05, ***P < 0.001).

To evaluate the impact of iron-overloaded macrophages on HSCs function, we examined HSCs cell viability, cell cycle distribution, and senescence in a co-culture system. CCK-8 assay results demonstrated a significant decrease in HSCs cell viability in the iron-overloaded group compared to the control group (*P* < 0.001) (Figure 4A). Flow cytometry analysis revealed that co-culture with THP-1-derived macrophages (HSCs + THP-1 + PMA) enhanced HSCs cycle progression, particularly increasing the G2/S phase population from 23.4 % to 27.1 % (*P* < 0.05). However, iron overload significantly hindered this progression, reducing the G2/S phase population to 18.2 % (*P* < 0.001) (Figure 4B). SA-β-gal staining indicated a notable reduction in senescence marker expression in the HSCs + THP-1 + PMA group compared to HSCs alone. In contrast, iron overload significantly elevated SA-β-gal expression, suggesting accelerated senescence (Figure 4C).

### 3.5 Mechanistic insights into HSCs dysfunction

**Figure 5:**
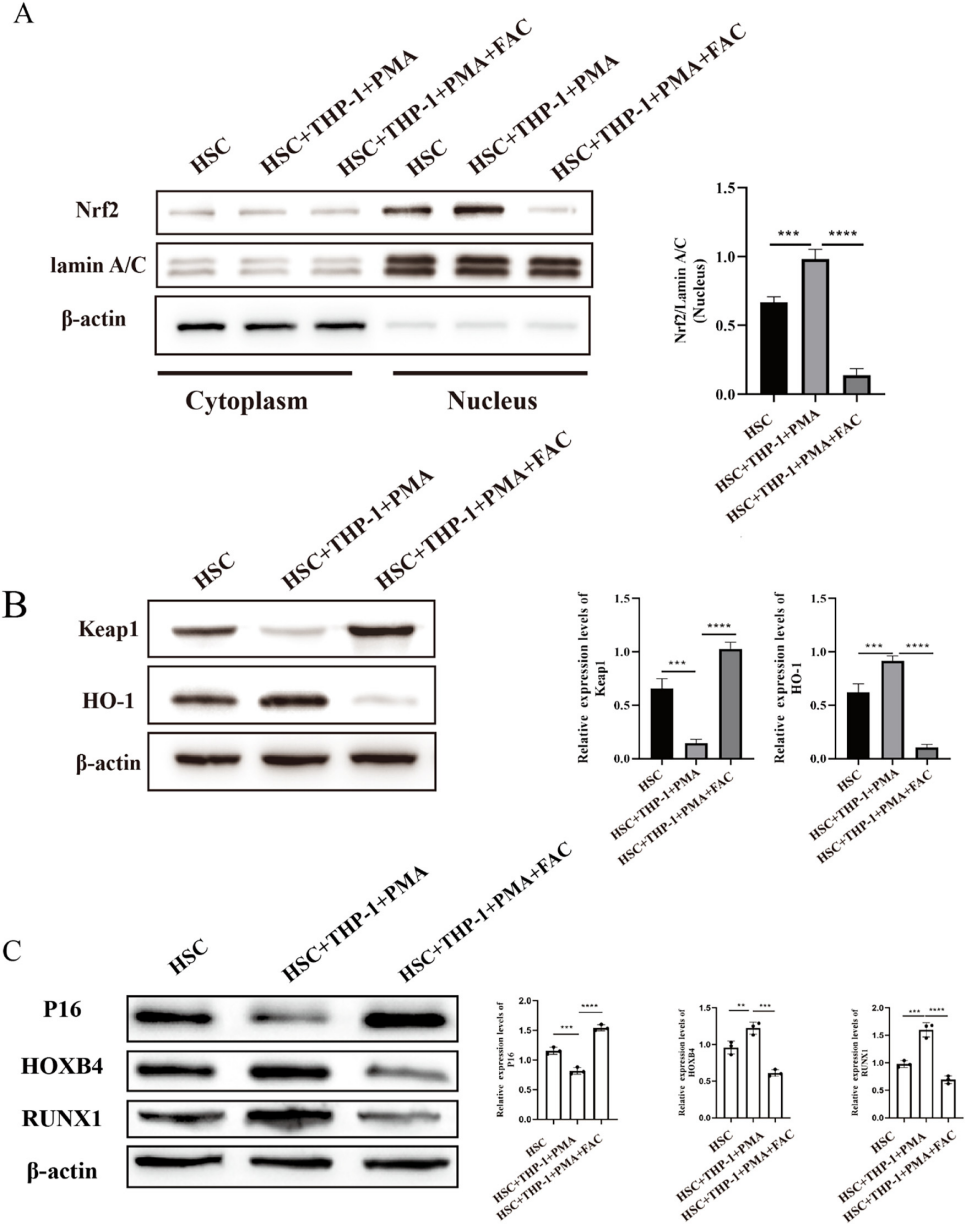
Iron overload-induced damage in HSCs by M0 macrophages. (A) Western blot analysis showing the levels of Nrf2 in both the cytoplasm and nucleus. Statistical analysis demonstrates Nrf2 nuclear expression in the various treatment groups. (B) Expression of Keap1 and HO-1 was measured by Western blot analysis in the different treatment groups. (C) Expression levels of P16, HOXB4, and RUNX1 were assessed using Western blot analysis in the various treatment groups (*n* = 3, **P < 0.01, ***P < 0.001, ****P < 0.0001).

To investigate the molecular mechanisms underlying HSCs impairment induced by iron overload, we examined key regulators of oxidative stress response and HSCs fate determination. Western blot analysis was conducted to assess the expression of the Nrf2/Keap1/HO-1 pathway. Nuclear Nrf2 levels were significantly elevated in the HSCs + THP-1 + PMA group compared to the HSCs-alone group (*P* < 0.001). However, in the iron overload group, Nrf2 expression was significantly reduced relative to the HSCs + THP-1 + PMA group (*P* < 0.0001) (Figure 5A). As shown in Figure 5B, Keap1 expression, which promotes Nrf2 degradation, was significantly downregulated in the HSCs + THP-1 + PMA group (*P* < 0.001) but was markedly upregulated under iron overload (*P*<*0.0001*) compared to the HSCs + THP-1 + PMA group. Similarly, HO-1 expression was significantly increased in the HSCs + THP-1+PMA group (*P* < 0.001) and decreased under iron overload (*P* < 0.0001) in comparison to the HSCs + THP-1 + PMA group. Furthermore, we analyzed HSCs fate markers via Western blot. In the HSCs + THP-1 + PMA group, the senescence marker P16 was significantly downregulated (*P* < 0.001), which was dramatically upregulated under iron overload (*P* < 0.0001) compared to the HSCs + THP-1 + PMA group. The self-renewal marker HOXB4 was significantly elevated in the HSCs + THP-1 + PMA group (*P* < 0.01), and it was substantially reduced under iron overload (*P* < 0.001). Additionally, the differentiation regulator RUNX1 was significantly increased in the HSCs + THP-1 + PMA group (*P* < 0.001), with a further marked upregulation under iron overload (*P* < 0.0001) (Figure 5C).

### 3.6 Phenotypic changes of Nrf2 activation on HSCs in a co-culture system

**Figure 6:**
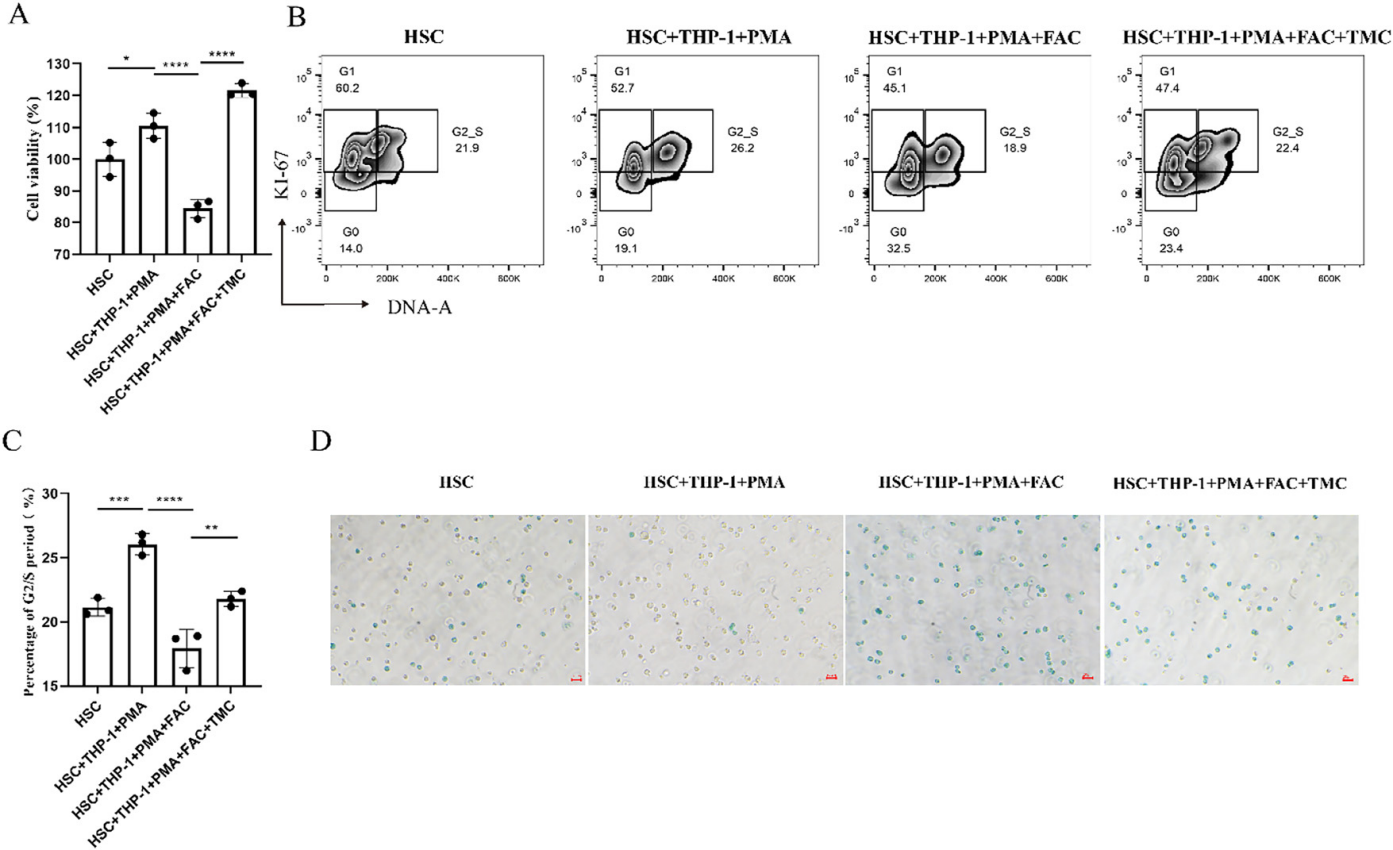
Phenotypic changes of Nrf2 activating by TMC in co-cultured system. (A) Cell viability was assessed by the CCK-8 assay in different treatment groups. (B–C) Cell cycle progression measured by flow cytometry analysis. Statistical analysis showing the percentage of G2/S period in different treatment group. (D) Results of different groups of SA-β-gal staining. Scale bar: 20 μm (*n* = 3; **P < 0.01, ***P < 0.001, ****P < 0.0001).

To explore the effects of Nrf2 activation on HSCs in a co-culture system, iron-overloaded M0 macrophages and HSCs were treated with the Nrf2 activator TMC. Cell viability was first assessed using the CCK-8 assay. Treatment with TMC significantly counteracted the inhibitory effects of iron overload on HSCs viability, resulting in a marked increase in viable cell compared to the HSCs + THP-1 + PMA + FAC group (*P* < 0.0001) (Figure 6A). Flow cytometry analysis further revealed that TMC treatment restored the proportion of HSCs in the G2/S phase, which was significantly higher than in the iron overload group (*P* < 0.01) (Figure 6B and C). Finally, TMC treatment significantly reduced SA-β-gal expression in the HSCs + THP-1 + PMA + FAC + TMC group, indicating a decrease in cellular senescence and a reversal of iron overload-induced senescence (Figure 6D).

### 3.7 Mechanistic insights into HSCs dysfunction treated by Nrf2 activation

**Figure 7:**
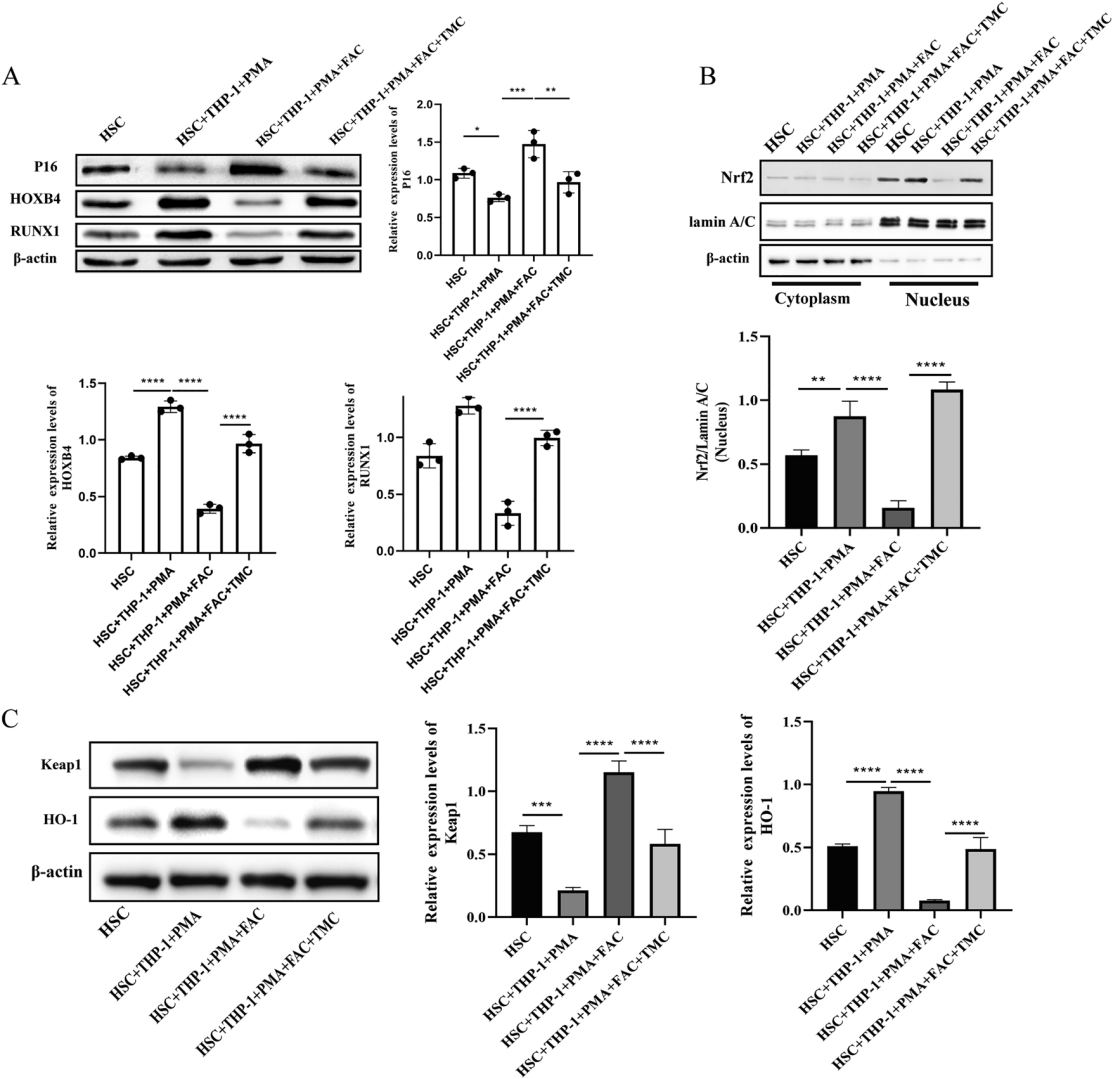
Nrf2 activation protects HSCs from iron overload-induced damage by M0 macrophages. (A) The P16, HOXB4 and RUNX1 expression was tested using Western blot assay in the different treatment group. (B/C) Nerf2, Keap1 and HO-1 expression measured by Western blot assay in the different treatment group (*n* = 3; *P < 0.05, **P < 0.01, ***P < 0.001, ****P < 0.0001).

To investigate the molecular mechanisms by which nuclear factor Nrf2 activation alleviates iron overload-induced dysfunction in HSCs, we found that in the HSCs + THP-1 + PMA + FAC + TMC group, compared with the HSCs + THP-1 + PMA + FAC group, the expression of P16 was significantly decreased (*P* < 0.01), while the levels of HOXB4 and RUNX1 were significantly increased (both *P* < 0.0001) (Figure 7A). We further analyzed the key proteins involved in the Nrf2/Keap1/HO-1 pathway. Western blot analysis showed that compared with the HSCs + THP-1 + PMA + FAC group, the expression of Nrf2 was significantly increased after TMC treatment (*P* < 0.0001) (Figure 7B). In addition, after TMC treatment, compared with the HSCs + THP-1 + PMA + FAC group, the expression of Keap1 was significantly decreased, while the expression of HO-1 was significantly upregulated (both *P* < 0.0001) (Figure 7C).

## 4 Discussion

This study systematically elucidates how iron-overloaded M0 macrophages disrupt HSCs homeostasis through perturbation of the Nrf2/Keap1/HO-1 signaling pathway. Iron-overloaded macrophages exhibited pronounced functional impairments, including reduced phagocytic activity, elevated ROS levels, and a heightened pro-inflammatory phenotype. These findings align with recent studies emphasizing that iron overload disturbs immune homeostasis; for example, imbalances in iron metabolism can affect macrophage polarization and T/B cell function [16], and iron overload can disrupt the immune-iron metabolism network, leading to immune cell dysfunction [17]. In a co-culture system, iron-overloaded macrophages significantly impaired HSCs proliferation and accelerated senescence by inhibiting Nrf2 nuclear translocation, upregulating Keap1, and downregulating HO-1 expression. Importantly, Nrf2 activation with TMC effectively reversed these damages, suggesting that targeting this pathway could be a potential therapeutic strategy for iron-related hematopoietic dysfunction.

We employed PMA-differentiated THP-1 macrophages as a model to study iron overload [18]. This model provides high experimental reproducibility, operational simplicity, and uniform responsiveness to stimuli, making it widely applicable for *in vitro* iron toxicity research. During FAC concentration screening, low-dose FAC (10–20 μM) increased calcein fluorescence, indicating a transient decrease in the LIP. Low concentrations of iron can rapidly induce ferroportin-1 expression and activation, promoting iron efflux to maintain cellular iron homeostasis [19]. Simultaneously, cells may upregulate ferritin heavy chain to sequester free iron, transiently lowering LIP [20]. These observations suggest that low-dose FAC triggers a rapid adaptive response centered on iron efflux and sequestration, producing temporary LIP reduction manifested as increased fluorescence. In contrast, at 160 μM FAC, these defense mechanisms were overwhelmed, resulting in rapid disruption of iron homeostasis, fluorescence quenching, and irreversible oxidative stress. However, this study did not include a group treated with FAC alone without PMA differentiation, which limits the ability to clearly distinguish the direct effects of iron overload from those associated with macrophage polarization. Future studies incorporating both undifferentiated and differentiated cell models, as well as comparative analyses of key functional markers such as iNOS and ROS signaling, will be necessary to more precisely define the independent and synergistic contributions of iron toxicity and polarization status.

Using a Transwell co-culture system, we further confirmed that iron-overloaded macrophages impair HSCs function through multiple mechanisms, including reduced viability, cell cycle arrest, and accelerated senescence. Clinically, patients with iron overload, such as transfusion-dependent thalassemia, often exhibit reduced HSCs numbers and impaired regenerative capacity. This damage is closely associated with iron-induced oxidative stress and the resultant ineffective hematopoiesis [21]. Iron overload also disrupts the microenvironment, weakening stromal support for HSCs; for example, impaired VCAM-1-mediated retention may result in HSPC loss or dysfunction from the niche [9]. Therefore, the inhibitory effect of iron-overloaded macrophages on HSCs likely reflects both intracellular iron imbalance and compromised microenvironmental support, emphasizing the importance of microenvironmental homeostasis for hematopoietic maintenance under iron overload.

This study further highlights Nrf2 as a central regulator of redox balance and stem cell protection. As a cellular stress sensor, Nrf2 mitigates oxidative damage by upregulating antioxidant genes such as HO-1 and NQO1 and modulating iron metabolism, countering iron-mediated toxicity. Although cell death was not the primary focus, iron-induced cytotoxicity and its potential effects on macrophage immune function merit attention. Excess iron can generate ROS via the Fenton reaction, cause mitochondrial dysfunction and lipid peroxidation, and destabilize multiple signaling pathways, exacerbating metabolic imbalance and immune cell dysfunction [22]. In our study, iron-loaded macrophages showed decreased phagocytic activity, elevated ROS, and increased iNOS expression, indicating significant immunoregulatory disruption linked to iron-dependent oxidative stress and inflammation. Ferroptosis, an iron-dependent form of regulated cell death, has emerged as a key player in immune regulation and disease pathogenesis [23]. While extensive cell death was not observed, sub-lethal ferroptosis signals, such as decreased GPX4 activity and lipid peroxide accumulation, may affect macrophage polarization and function. Low-level ferroptotic stress can drive macrophages toward pro-inflammatory polarization and metabolic reprogramming, which may compromise HSCs support [24]. Collectively, these findings suggest that iron overload disrupts hematopoietic microenvironment homeostasis through both oxidative stress and sub-lethal ferroptosis mechanisms. Notably, TMC-mediated Nrf2 activation restored HO-1 expression and reversed impaired HSCs proliferation and accelerated senescence, highlighting Nrf2 as a key protective regulator for both macrophages and stem cell homeostasis. Future studies using iron chelators, ferroptosis inhibitors (e.g., ferrostatin-1), and genetic manipulation will clarify the precise roles of ferroptosis and iron toxicity in macrophage dysfunction, offering potential therapeutic targets.

Despite providing mechanistic insights, several limitations should be noted. M0 macrophages derived from THP-1 cells, while experimentally controllable, cannot fully recapitulate the heterogeneity and tissue-specific characteristics of primary human macrophages. Macrophages from different sources (e.g., bone marrow-derived or hepatic Kupffer cells) may vary in iron-handling capacity, cytokine secretion, and signal transduction. The Transwell system reveals paracrine interactions but cannot reproduce the complex spatial architecture and extracellular matrix signals of the native bone marrow niche. The precise mechanisms by which iron-overloaded macrophages inhibit Nrf2 activity in HSCs remain unclear and may involve oxidative mediators, extracellular vesicles, or direct contact. Addressing these questions requires primary cell models, conditional genetic manipulation, and advanced 3D co-culture or *in vivo* studies.

In summary, this study elucidates how iron-overloaded macrophages disrupt HSCs homeostasis via the Nrf2/Keap1/HO-1 pathway and demonstrates that Nrf2 activation can mitigate these effects. These findings underscore the interplay between iron metabolism and the hematopoietic microenvironment and provide a framework for developing novel strategies to treat iron-related hematopoietic disorders. Further investigation of ferroptosis signaling, intercellular communication, and microenvironmental structure will facilitate translation from mechanistic insight to clinical application.

## 5 Conclusions

This study underscores the role of iron overload in disrupting M0 macrophage redox balance, driving inflammatory polarization, and impairing HSCs function. Furthermore, activating the Nrf2/Keap1/HO-1 axis may offer a therapeutic strategy for iron overload-associated hematopoietic disorders. Future research should explore combinatorial therapies targeting both iron chelation and Nrf2 activation, guided by recent advancements in ferroptosis-immunotherapy crosstalk.
